# Outcomes of COVID-19 in Solid Organ Transplants

**DOI:** 10.7759/cureus.11344

**Published:** 2020-11-05

**Authors:** Saritha Ranabothu, Swetha Rani Kanduri, Krishna Nalleballe, Wisit Cheungpasitporn, Sanjeeva Onteddu, Karthik Kovvuru

**Affiliations:** 1 Pediatrics, University of Arkansas for Medical Sciences, Little Rock, USA; 2 Nephrology, Ochsner Medical Center, New Orleans, USA; 3 Neurology, University of Arkansas for Medical Sciences, Little Rock, USA; 4 Nephrology, University of Mississippi Medical Center, Jackson, USA

**Keywords:** covid 19, patient outcomes, solid organ transplant, transplant

## Abstract

The novel coronavirus disease 2019 (COVID-19) is a global pandemic affecting millions of people worldwide. Solid organ transplant (SOT) recipients are probably at higher risk of severe infection and associated complications from COVID-19. Data on clinical outcomes of COVID-19 infection in SOT recipients are limited. Using the TriNetX database, patients with laboratory-confirmed COVID-19 from January 20, 2020, to July 7, 2020, were included in the study. We compared clinical outcomes comprising hospitalization, need for critical care services, intubation, and mortality among SOT recipients and patients without SOT. Of 30,573 laboratory-confirmed COVID-19 patients, 288 had SOT. Patients with SOT were more likely to be hospitalized (37.2% vs. 12.2%; p < 0.0001), needed critical care services (6.9% vs. 2.3%; p < 0.0001), needed intubation (7.9% vs. 2.0%; p < 0.0001), and had a higher 30-day mortality (11.1% vs. 3.8%; p < 0.0001). Patients in the transplant group were older (55.4 vs. 47.6 years; p < 0.0001) and had a higher prevalence of medical co-morbidities. SOT recipients are at significant risk of adverse COVID-19 related outcomes, including hospitalization, need for critical care services, and 30-day mortality, likely due to multiple co-morbid conditions.

## Introduction

The ongoing pandemic of the novel coronavirus disease 2019 (COVID-19) infection has implied a tremendous health hazard on the world population. So far, approximately 13 million people are infected with COVID-19, leading to 565,000 reported deaths and we continue to face significant challenges [1]. COVID-19 infection caused by SARS-CoV-2 (severe acute respiratory syndrome coronavirus 2) has not only posed tremendous mortality and morbidity risk but also impacted financial, social, and emotional aspects of human well-being [1]. Solid organ transplant (SOT) recipients presumably are at high risk for COVID-19 infection, especially elderly SOT recipients aged > 60 years [2,3]. Low threshold for testing COVID-19 infection in transplant population is probably beneficial as they manifest atypical symptoms, predominantly diarrhea [4]. The spectrum of clinical disease varies from asymptomatic viral infection to life-threatening illness necessitating mechanical ventilation and renal replacement therapies [2,5]. COVID-19 infection is associated with elevated levels of cytokines, particularly IL-6 (interleukin-6), from an exaggerated host immune response [6,7]. In determining the course of disease, cellular immunity plays a notable role, placing SOT recipients at potential risk of severe complications from COVID-19 infections [8].

Earlier reports suggested similar clinical courses and favorable outcomes in SOT recipients compared with non-SOT population, likely secondary to attenuated immune and cytokine responses [9-11]. Heterogeneous clinical course among SOT cohort, with potential of rapid progression to complicated and fatal clinical course of acute respiratory distress syndrome, has also been reported [12]. Recent studies have reported severe clinical course and worse outcomes while on immunosuppression, with associated co-morbidities [4,13-14]. Given the disparities in existing data, we conducted this study to compare the outcomes among SOT recipients and non-SOT population with COVID -19 infection.

## Materials and methods

TriNetX, a world health collaborative clinical research database that facilitates collecting real-time electronic medical record data from the network of health care organizations across the United States and internationally, was used for extracting information about de-identified COVID -19 patients with and without SOT. “COVID-19 Research Network” in TriNetX represents an extremely large, worldwide COVID-19 database.

While the TriNetX database does not acknowledge data downloads or individual patient information for review, it authorizes analysis in the form of queries. Queries made through the TriNetX database operate through browser and real-time features. Several studies on COVID -19 were published to date using the TriNetX database [15-19] and have been described in detail elsewhere [20]. At the University of Arkansas for Medical Sciences, Arkansas Clinical Data Repository and Department of Biomedical Informatics manage TriNetX data.

Study protocol

Study protocol was implemented after appropriate approval was obtained from the Institutional Review Board. The analysis was conducted on July 7, 2020, on the TriNetX COVID-19 Research Network. We identified SOT patients using the following ICD-10 (the International Classification of Diseases, Tenth Revision) codes: kidney transplant (Z94.0), heart transplant (Z94.1), lung transplant (Z94.2), and liver transplant (Z94.4) [21]. Patients with laboratory-confirmed COVID-19 on or after January 20, 2020, to July 7, 2020, were identified using the LOINC (Logical Observation Identifiers Names and Codes) code (9088: SARS coronavirus 2 and related RNA positive). Thereafter, baseline demographics, co-morbidities, and clinical outcomes, including hospitalization, intubation, need for critical care services, and mortality within 30 days from diagnosis of COVID-19, were compared in patients with and without SOT. TriNetX analytics function was utilized in performing statistical analysis. Outcomes were analyzed using Z-test, propensity score matching (demographics and co-morbidities) was conducted using a t-test. Descriptive statistics were reported as number of observations and percentage or as mean ± standard deviation, as applicable. Two physicians (K. N. and S. O.) independently performed these queries.

## Results

A total of 288 SOT patients were identified using the TriNetX database, with laboratory-confirmed COVID-19 between January 20, 2020, and July 7, 2020. Among them, 109 (37.8%) patients were females, 89 (30.9%) patients were Caucasians, 83 (28.8%) African-Americans, 37 (12.8%) were Hispanic, and 79 (27.4%) were of other races/ethnicities/unknown. There were 30,285 laboratory-confirmed COVID-19 patients without SOT in the database. Baseline characteristics such as race, gender, and co-morbidities in the transplant versus non-transplant groups are detailed in Table 1.

**Table 1:** Baseline demographics and co-morbidities of SOT and non-SOT patients with COVID-19. SOT, solid organ transplant

	SOT patients	Non-SOT patients	p-Value
Number of patients	288	30,285	NA
Age (years)	55.4 ± 15	47.6 ± 20.3	<0.0001
Female	109 (37.8%)	15,913 (52.5%)	<0.0001
Race			
Caucasian	89 (30.9%)	11,187 (36.9%)	0.0346
African American	83 (28.8%)	5,715 (18.8%)	<0.0001
Hispanic (ethnicity)	37 (12.8%)	4,566 (15.1%)	0.2923
Co-morbid conditions			
Hypertension	262 (90.9%)	7,236 (23.9%)	<0.0001
Diabetes mellitus	164 (56.9%)	3,841 (12.7%)	< 0.0001
Ischemic heart disease	134 (46.5%)	2,147 (7.1%)	<0.0001
Chronic kidney disease	237 (82.3%)	1,516 (5.0%)	<0.0001
Heart failure	110 (38.2%)	1,527 (5.0%)	<0.0001
Atrial fibrillation and flutter	64 (22.2%)	1,194 (3.9%)	<0.0001
Obstructive pulmonary disease	46 (15.9%)	1,149 (3.8%)	<0.0001
Overweight and obesity	114 (39.6%)	4,358 (14.4%)	<0.0001
Nicotine dependence	41 (14.2%)	1,873 (6.2%)	<0.0001
Ischemic stroke	16 (5.6%)	690 (2.3%)	0.0002

Among the two groups, patients with transplant were older (55.4 vs. 47.6 years; p < 0.0001) and had a significantly higher prevalence of co-morbidities including diabetes mellitus, ischemic heart disease, hypertension, smoking, obesity, chronic kidney disease, chronic obstructive pulmonary disease, heart failure, atrial fibrillation, and prior ischemic stroke (p < 0.0001 except for ischemic stroke for which p = 0.0002) (Table 1). When comparing outcomes, analysis showed statistically significant higher need for hospitalization (37.2% vs. 12.2%; p < 0.0001) critical care services (6.9% vs. 2.3%; p < 0.0001), intubation (7.9% vs. 2.0%, p < 0.0001), and higher 30-day mortality (11.1% vs. 3.8%; p < 0.0001) in the SOT group (Table 2).

**Table 2:** Comparison of outcomes among SOT and non-SOT patients with COVID-19. Note: 95% confidence interval provided within bracket for each odds ratio. SOT, solid organ transplant; NA, not applicable

	SOT patients	Non-SOT patients	p-Value	Odds ratio
Number of patients	288	30,285	NA	NA
Clinical outcome				
Hospitalization	107 (37.2%)	3,698 (12.2%)	<0.0001	4.25 (3.34-5.41)
Critical care services	20 (6.9%)	690 (2.3%)	<0.0001	3.20 (2.02-5.07)
Intubation	23 (7.9%)	621 (2.0%)	<0.0001	4.15 (2.69-6.39)
Mortality	32 (11.1%)	1,155 (3.8%)	<0.0001	3.15 (2.17-4.57)

Among the 288 SOT patients, 224 were kidney transplant patients. In these 224 patients, 88 (39.3%) patients needed hospitalization, 15 (6.7%) patients needed critical care services, 20 (8.9%) patients needed intubation, and 30-day mortality was seen in 21 (9.4%) patients (Table 3). As a small number of patients with liver, lung, and heart transplant acquired COVID-19, meaningful conclusions regarding outcomes could not be ascertained.

**Table 3:** Subgroup analysis of outcomes among COVID-19 infected kidney transplant patients.

	Kidney transplant patients
Number of patients	224
Clinical outcome	
Hospitalization	88 (39.3%)
Critical care services	15 (6.7%)
Intubation	20 (8.9%)
30 day- mortality	21 (9.4%)

Among these 288 patients, 61 (21%) were from North East, 62 (22%) from Midwest, 56 (19%) from West, 46 (16%) from South of the US, and 63 (22%) from outside the US.

## Discussion

Current literature on SOT recipients with COVID infection is dynamic and rapidly evolving. Our study is unique and one of the largest reported thus far, and includes 288 SOT recipients with COVID infection compared with 30,285 COVID positive non-SOT population. While some early small case series reported favorable outcomes among SOT recipients during their initial clinical course [12,22], large studies with longer follow-ups reported greater adverse events [14,23]. In the study by Pereira et al. [14], which included 90 SOT recipients with COVID-19 infection in New York City during a three-week period, 76% of patients were hospitalized and 34% required ICU, with 18% overall mortality (52% ICU mortality). A recent international study among COVID infected kidney transplant recipients, with a 52-day mean follow-up, reported 32% mortality among hospitalized patients and 52% mortality among patients in ICU, with a median time to death of 15 days [23]. As discussed in the Results section, our study reaffirms that COVID like any other infection causes adverse outcomes in SOT recipients and that the earlier thoughts of attenuated immune response potentially offering protection from cytokine storm is probably not true. Our results, similar to recently published reports by Fernández-Ruiz et al. [4] and Akalin et al. [13], emphasize that associated co-morbidities of SOT recipients probably contribute to increased vulnerability, as well as unpredictable and fatal clinical course.

There are some intrinsic limitations to our study, and some of them are inherent to large databases. First, this was a retrospective study and the data were obtained based on ICD 10-coding, which could be subjected to reporting bias. Second, it remains unclear whether adverse outcomes are secondary to severe COVID infection versus an effect of organ dysfunction among transplant patients. Third, subgroup analysis and outcomes among patients with heart, liver, and lung transplant could not be provided given the very limited data to make any analysis. Last, participants could have false-positive results on how COVID-19 is tested, and such limitations exist in the majority of reports on COVID-19 during an ongoing pandemic.

## Conclusions

Our study depicted worse outcomes with an increased need for hospitalization among COVID-19 infected SOT recipients when compared with COVID-19 infected non-SOT population likely due to the prevalence of multiple co-morbid conditions in SOT recipients, suggesting that SOT recipients should receive utmost care to help prevent this dreadful infection and early referral to transplant centers with a higher level of care. Spreading awareness and education regarding preventive measures against COVID-19 infection are crucial in providing protection to these immunologically challenged populations. Implementing telehealth might help to curb some exposure risk.
